# Supplementary figures and images for: Sequencing and Characterization of M. morganii Strain UM869: A Comprehensive Comparative Genomic Analysis of Virulence, Antibiotic Resistance, and Functional Pathways
Source: Genes (Basel). 2023 Jun 16;14(6):1279. doi: 10.3390/genes14061279 (PMC10298637; doi:10.3390/genes14061279)

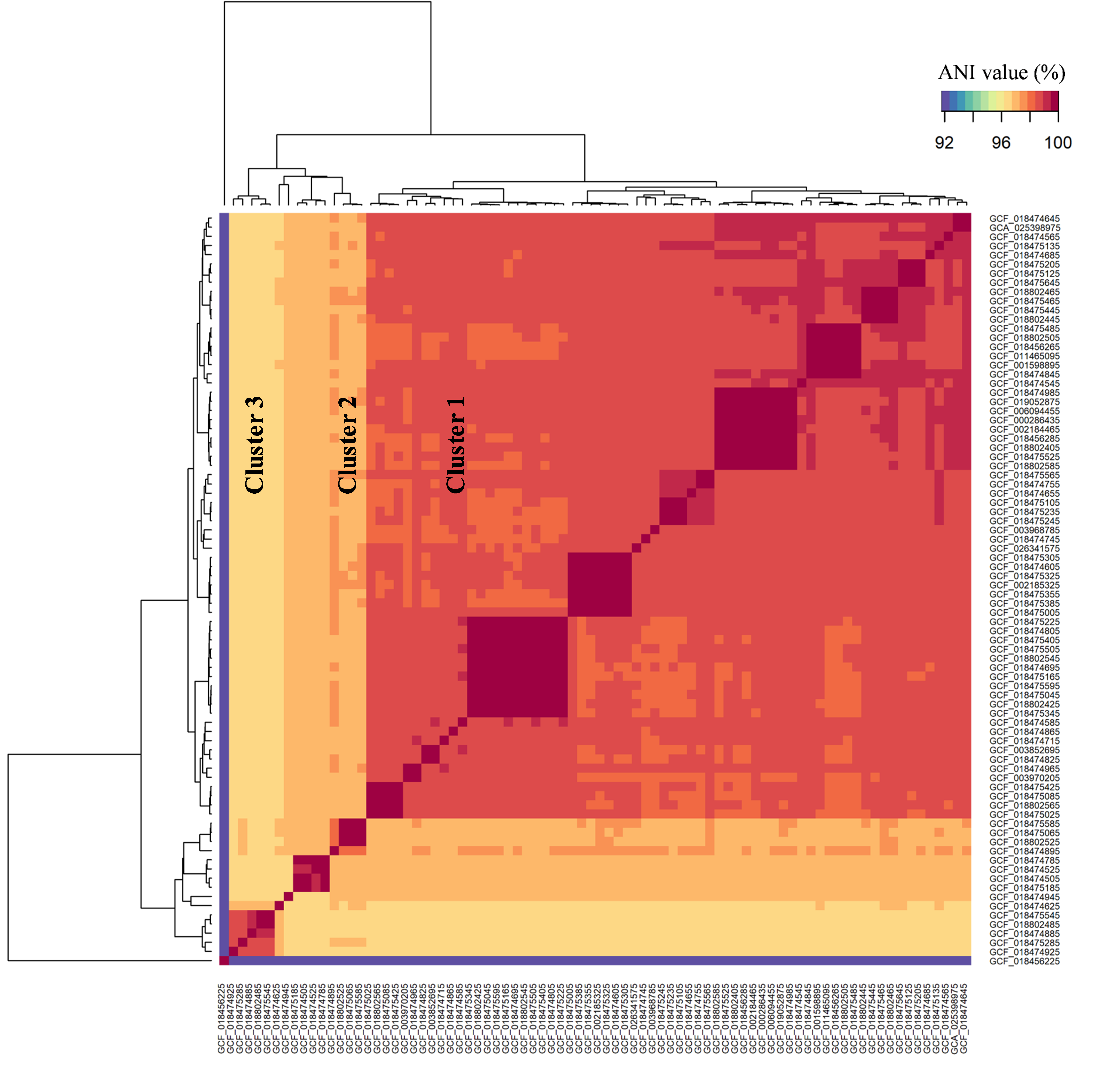

Supplement: Supplementary file 1 [file genes-14-01279-s001.zip › Figure_S2_Revised.tif]
